# Declines in body size of sockeye salmon associated with increased competition in the ocean

**DOI:** 10.1098/rspb.2022.2248

**Published:** 2023-02-08

**Authors:** Jan Ohlberger, Timothy J. Cline, Daniel E. Schindler, Bert Lewis

**Affiliations:** ^1^ School of Aquatic and Fishery Sciences, University of Washington, Seattle, WA 98195, USA; ^2^ Department of Ecology, Montana State University, Bozeman, MT, 59717, USA; ^3^ Alaska Department of Fish and Game, Commercial Fisheries Division, Anchorage, AK 99518, USA

**Keywords:** age structure, body size, size-at-age, sockeye salmon, climate change

## Abstract

Declining body sizes have been documented for several species of Pacific salmon; however, whether size declines are caused mainly by ocean warming or other ecological factors, and whether they result primarily from trends in age at maturation or changing growth rates remain poorly understood. We quantified changes in mean body size and contributions from shifting size-at-age and age structure of mature sockeye salmon returning to Bristol Bay, Alaska, over the past 60 years. Mean length declined by 3%, corresponding to a 10% decline in mean body mass, since the early 1960s, though much of this decline occurred since the early 2000s. Changes in size-at-age were the dominant cause of body size declines and were more consistent than trends in age structure among the major rivers that flow into Bristol Bay. Annual variation in size-at-age was largely explained by competition among Bristol Bay sockeye salmon and interspecific competition with other salmon in the North Pacific Ocean. Warm winters were associated with better growth of sockeye salmon, whereas warm summers were associated with reduced growth. Our findings point to competition at sea as the main driver of sockeye salmon size declines, and emphasize the trade-off between fish abundance and body size.

## 1. Introduction

Body size has fundamental effects on many aspects of the life history and ecology of organisms, and it changes throughout life due to ontogenetic growth and development [1–3]. Age and size distributions are genetically determined and modulated at the population level by environmental factors such as climate, variation in population density, and species interactions related to competition and predation. Population size and age distributions change in response to size-selective harvest and via environmental effects on individual growth and size-dependent survival. Climate warming has been suggested to cause widespread declines in organism body size [4,5], especially in aquatic ectotherms such as fishes ([6]; but see [7]). In addition, intense harvesting can lead to age-truncated or juvenescent populations, especially when fisheries selectively remove large fish [8,9] and cause evolutionary changes in life-history traits resulting in altered growth and maturation schedules [10,11].

Recent studies on Pacific salmon (*Oncorhynchus* spp.) suggest that shifting demographic structures, which have resulted in declines in the mean body size of mature salmon since at least the 1970s, are linked to changes in climate and increased competition in the ocean [12,13]. Declines in salmon body size can have negative repercussions for fishery yield and other ecosystem services, for instance by reducing the per capita reproductive output or by decreasing the commercial value for harvesters [13,14]. Observed changes in body size of salmon that return to freshwater habitats to spawn can result from a combination of two proximate causes, from fish maturing at younger ages (age structure shift) or smaller size at a given age of mature fish (size-at-age shift) [13,15]. Ecological and evolutionary processes affecting maturation timing in salmon mainly manifest as age structure shifts in adults whereas processes affecting growth mainly manifest as shifts in size-at-age; however, because maturation in salmon depends on size and age, changes in growth rate can also trigger changes in maturation schedules [16,17]. Understanding the proximate and ultimate causes of changes in body size can help reveal the underlying mechanisms of size declines and inform future management.

We quantified changes in the body size and age structure of sockeye salmon (*O. nerka*) in Bristol Bay, Alaska, one of the most productive sockeye salmon regions in the world with an average annual return of about 35 million fish over the past 60 years (electronic supplementary material, figure S1). Bristol Bay sockeye salmon are harvested in a commercial fishery that represents roughly half of the global annual catch of this species. Fish that escape harvest in the fishery and spawn in the major river systems that drain into Bristol Bay are enumerated each year such that run sizes for individual stocks are estimated with high accuracy. Using data collected from in-river monitoring projects and from the commercial fishery, in combination with estimates of total catch and escapement (i.e. fish that pass through the fishery) in each river, we reconstructed age and length information of individual sockeye salmon that have returned to seven of the nine major river systems of Bristol Bay in the years 1963 to 2020. This reconstructed dataset contained roughly 2 billion individuals. We quantified changes in mean length over time and estimated contributions of trends in size-at-age and age structure to observed changes in mean body size. We also evaluated evidence for the effects of climate as well as intra- and interspecific competition in the ocean (using salmon abundances in the ocean as a proxy for the strength of competition) on changes in size-at-age to understand the mechanisms responsible for declining size in Bristol Bay sockeye salmon. Changing ocean temperatures can affect size-at-age via direct effects on fish bioenergetics and thus growth, or via ecological interactions affecting growth potential or size-dependent survival. Intra- and interspecific competition for shared prey resources can cause size changes via food-dependent growth. Finally, intense size-selective harvesting can cause long-term evolutionary changes in traits related to growth or maturation that result in shifting size-at-age of mature fish.

## 2. Methods

### (a) Species and region

#### (i) Bristol Bay

**Figure 1.**
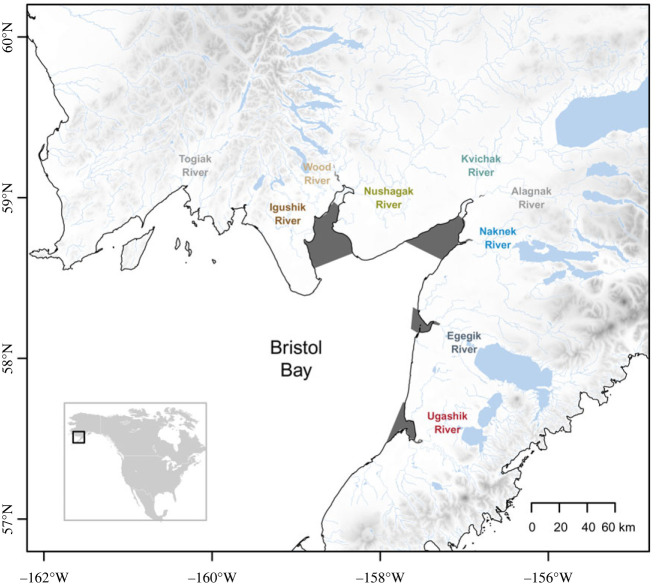
Map of the Bristol Bay region. The map shows Bristol Bay and its major sockeye salmon-producing river systems: Togiak, Igushik, Wood, Nushagak, Kvichak, Alagnak, Naknek, Egegik and Ugashik (river systems not used in the analysis in light grey font). Dark grey areas indicate commercial fishing districts, grey shading on land indicates elevation and the inset at the bottom left indicates the location of the main map.

Bristol Bay in southwest Alaska is the southeastern region of the Bering Sea. The nine major river systems that flow into Bristol Bay are Togiak, Igushik, Wood, Nushagak, Kvichak, Alagnak, Naknek, Egegik and Ugashik rivers (figure 1). The analyses presented here include data for seven of the nine major river systems that together account for an average of more than 90% of the annual sockeye salmon returns to Bristol Bay (electronic supplementary material, figure S1). The Togiak and Alagnak rivers were not included due to insufficient age–length (AL) data for the escapement over extended periods during the past 60 years. Besides sockeye salmon, four other species of Pacific salmon also spawn and rear in the watersheds of Bristol Bay: Chinook salmon (*O. tshawytscha*), coho salmon (*O. kisutch*), chum salmon (*O. keta*) and pink salmon (*O. gorbuscha*), though at considerably lower abundances [18]. Salmon support diverse wildlife, human culture and livelihoods in the region through subsistence, sport and commercial fisheries.

#### (ii) Sockeye salmon ecology

Sockeye salmon are anadromous fish that incubate as embryos and then hatch in freshwater habitats, typically spend 1 or 2 years rearing in freshwater lakes, migrate to the ocean where they typically spend 2–3 years to attain most of their body growth before returning to freshwater ecosystems to spawn and then die. Across the river systems considered here, roughly 99% of all fish that return to Bristol Bay belong to one of four dominant age groups: 1.2, 1.3, 2.2 or 2.3 (where the first number indicates years spent as free-swimming juveniles in fresh water and the second number indicates years spent in the ocean). The oldest dominant age group (2.3) is 6 years of total age: 1 as an embryo in the gravel, 2 in freshwater and 3 in the ocean.

### (b) Sockeye salmon data

#### (i) Age–length sampling and enumeration

The Alaska Department of Fish & Game (ADF&G) monitors the numbers, ages and lengths of sockeye salmon returning to the major river systems of Bristol Bay. AL data have been collected annually from commercial harvests and escapement since the early 1960s. AL sampling from commercial drift and set gill net harvests occurs in marine waters and near river mouths. Fish in spawning populations (escapement) are monitored at counting towers and a sonar site (Nushagak River) where fish numbers are estimated and AL data are collected. At escapement monitoring sites, fish are collected primarily using beach seines (drift gill nets are used on the Nushagak River). AL datasets are collected using standardized methods following Tobias *et al*. [19]. Fish length was measured as mid-eye-to-fork length to the nearest millimetre. Fish age was estimated from scale pattern analysis with standardized scale collection and ageing protocols. Circular growth patterns on sockeye salmon scales differ between freshwater when growth is relatively slow and the ocean when growth increases rapidly, as well as between summer and winter months (widely versus narrowly spaced circular rings). This scale pattern allows assigning fish to freshwater and ocean age groups. The AL dataset contained roughly 2.8 million individual AL samples, with average annual sample sizes of 28 000 and 19 000 fish from the catch and escapement, respectively. AL samples are representative of the sizes of fish in the catch and escapement for each river in each year. However, AL data in any given year are not sampled proportional to the run abundance in each river and not sampled proportional to the relative numbers of fish in the catch and escapement, thus using raw AL samples would lead to biased estimates of river-specific and bay-wide mean sizes.

#### (ii) Age and length reconstructions

To reconstruct size and age information for all sockeye salmon returning to the seven river systems of Bristol Bay examined here, we resampled size and age information collected in AL sampling according to return-by-age information contained in the brood table constructed for each river (C. Cunningham 2021, personal communication). Brood tables apportion the fish that return in a given calendar year to age classes for each previous brood year (i.e. the year their parents spawned) in each river. They are estimated from run reconstructions that account for AL sampling data from each fishing district as well as genetic stock identification of fish in the harvest and total returns to each river [20]. We assigned AL data collected by return year to their respective brood year based on total age. For each brood year, river and age group, we randomly resampled individuals from AL samples collected in the escapement according to the total number of individuals for that year, river and age group in the brood table. We repeated this procedure for AL samples collected from commercial harvest within fishing districts. We did not attempt to separate river-specific AL data from within districts due to the lack of suitable methods to allocate catch samples to natal rivers. Resampled individuals from the escapement were combined with the resampled individuals from harvest to generate complete AL information for each river and brood year. This reconstructed dataset contained information for roughly 2 billion fish.

### (c) Climate and salmon abundance data

This section introduces the environmental and ecological variables used to test for effects of climate and competition on sockeye salmon size, including fresh water and ocean temperatures, as well as the abundances of sockeye salmon and the competitor species pink and chum salmon. Ocean temperature data were obtained from the NCEP/NCAR reanalysis project by the NOAA Earth System Research Laboratory [21]. Based on previous work on the ocean distributions of Bristol Bay sockeye salmon [22,23], temperature data were averaged over four spatial grids representing temperature conditions in Bristol Bay (56.2–60°N 157.5–163.1°W), the southern Bering Sea (54.3–60°N 165–180°W), the north-western Gulf of Alaska (46.7–52.4°N 157.5–172.5°W) and an area north and south of the Aleutian Islands (50.5–58.1°N 159.4–180°W) (electronic supplementary material, figure S2). We used seasonal average sea surface temperatures (SSTs) for winter (January–March) and summer (July–September). Freshwater temperature records were obtained from Lake Aleknagik in the Wood River system [12]. Lake temperatures from the top 20 m were averaged using bi-weekly measurements for the period 15 June–5 September. By using this time series, we assumed that the year-to-year variation in average summer temperatures in Lake Aleknagik was indicative of the freshwater temperature conditions in the Bristol Bay region. Finally, total abundances of Asian and North American pink (*O. gorbuscha*) and chum salmon (*O. keta*) in the North Pacific Ocean were obtained from Ruggerone and Irvine [24] for 1960–2015 and from Ruggerone *et al*. [25] for 2016–2020. Pink salmon abundances were included to account for competition because previous work suggested negative effects of pink salmon on sockeye salmon body size [26,27].

### (d) Statistical analyses

Based on reconstructed AL information for sockeye salmon returning to Bristol Bay, we estimated trends in mean body size since the early 1960s, and contributions to size trends from changes in size-at-age and age structure, for each of seven river systems and bay-wide. All AL observations are for fish that have matured and are returning from the ocean to reproduce in freshwater habitats. Size-at-age of immature fish is different, because maturation in salmon depends on size and age, and slower growing individuals tend to mature later than fast growing individuals of the same cohort. In this paper, we refer to ‘mean size’ and ‘mean size-at-age’ instead of using ‘mean size-at-maturation’ and ‘mean size-at-age-at-maturation’, respectively.

#### (i) Mean size

The following calculations were performed by brood year to avoid confounding of mean size trends with temporal trends in recruitment. Variation in year-class strength translates into variation in age composition and thus variation in mean size when assessed on a return year basis [28], whereas age compositions and mean size are unaffected by recruitment variation when assessed on a brood year basis. While our dataset covers all return years until 2020, the last brood year included in the analysis was 2014 to ensure that all dominant age groups from that brood year have been observed in the return.

Based on the size (*S*) of all individuals (*i*) across age groups (*a*) in the reconstructed dataset and the number of fish (*N_y_*_,*r*_) in a given brood year (*y*) and river (*r*), we calculated river-specific annual mean body sizes (${\bar{S}}_{y,r}$):

$${\bar{S}}_{y,r}=\frac{\sum_{a}\sum_{i}S_{i,a,y,r}}{N_{y,r}}.$$

Accordingly, Bristol Bay wide mean body size in a given year was calculated as the sum of all individuals across age groups and rivers:

$${\bar{S}}_{y}=\frac{\sum_{r}\sum_{a}\sum_{i}S_{i,a,y,r}}{N_{y}},$$

where *N_y_* is the total number of fish from brood year *y*.

Mean body size (length, mm) in each river and bay-wide was calculated for the total return, i.e. all fish in the catch and escapement that have returned from a given brood year.

Mean length was converted to mean mass using a length–weight relationship of the form:

$$W=aS^{b},$$

where *W* is body size measured in grams mass, *S* is body size measured in millimetres length, *a* is the allometric scalar and *b* is the allometric exponent. The relationship was fit using nonlinear least-squares regression. We used samples from the commercial catch in 2010–2015 across age groups and districts (*N* = 15 100) for which mass and length data were available. This conversion does not account for spatial or temporal variation in the length–weight relationship. It was performed to provide an estimate of changes in body mass over time at the bay-wide scale.

#### (ii) Mean size-at-age anomalies

We calculated individual size-at-age anomalies as the difference between an individual's size and the long-term mean size of its age group in a given river system (electronic supplementary material, figure S3):

$$SAA_{i,a,y,r}=S_{i,a,y,r}-{\bar{S}}_{a,r},$$

where *S_i_*_,*a*,*y*,*r*_ is the size of individual *i* in age group *a*, year *y* and river *r*, and ${\bar{S}}_{a,r}$ is the long-term mean size of that age group and river:

$${\bar{S}}_{a,r}=\frac{\sum_{y}\sum_{i}S_{i,a,y,r}}{N_{a,r}},$$

where *N_a_*_,*r*_ is the number of fish in age group *a* and river *r*. Individual size-at-age anomalies are expressed in unit lengths (mm) on an absolute scale and can therefore be aggregated across age groups to calculate a mean annual size-at-age anomaly, which is the mean of all individual size-at-age anomalies in a given year and river:

$${\bar{SAA}}_{y,r}=\frac{\sum_{a}\sum_{i}SAA_{i,a,y,r}}{N_{y,r}}.$$

Annual mean size-at-age was highly correlated among age groups with an average pairwise correlation of 0.7 bay-wide (electronic supplementary material, figure S4). Annual size-at-age anomalies were computed by brood year to calculate contributions to changes in mean size and were also computed by return year to model covariate effects on size-at-age (see section ‘*Covariate models of changes in size-at-age*’).

Accordingly, the mean annual size-at-age anomaly for Bristol Bay (${\bar{SAA}}_{y}$) was calculated as the average of all individual size-at-age anomalies across age groups and calculated as the difference between an individual's size and the long-term mean size of its age group across river systems.

Because the reconstructed AL dataset represents the complete reconstruction for the population in each river, mean size and size-at-age metrics of the return are intrinsically weighted by catch versus escapement numbers within populations, and by the relative abundances of each population when calculating bay-wide metrics. The mean size-at-age anomaly provides a way to quantify the contribution of trends in size-at-age to changes in mean body size over a given time period.

#### (iii) Contribution of changes in size-at-age and age structure to mean size trends

To quantify the contribution of trends in size-at-age relative to the contribution of trends in age structure to the changes in overall mean body size, we calculated the mean size and mean size-at-age anomaly during each of the first and last 5 years of the time series and used the difference between the late and early period as our metric of total size change. Because the mean size-at-age anomaly is also in unit length (mm), changes over time can be compared directly to those in mean body size. We also evaluated the temporal development of changes in the sizes of Bristol Bay sockeye salmon by comparing the mean body size and size-at-age differences between the first 5 years of the record, and all subsequent rolling 5-year periods. Alternative approaches for estimating trends over time, such as fitting a linear regression model that incorporates autoregressive errors and model weights based on total abundance in each year, resulted in similar estimates that did not change our general conclusions. The difference between changes in mean size and mean size-at-age anomaly is the contribution from shifting age structure, which has changed considerably over time (electronic supplementary material, figure S4).

#### (iv) Covariate models of changes in size-at-age

We fit covariate models to explain variation in the mean size-at-age anomaly of Bristol Bay sockeye salmon by return year using least-squares linear regression. We therefore repeated the above calculations by return year instead of brood year to get annual estimates of the average size-at-age of fish returning in a given calendar year. The predictors considered in the models were the total run abundance of Bristol Bay sockeye salmon, the abundance of pink salmon and chum salmon in the North Pacific, fishery selection differentials, freshwater rearing temperature and mean SST in the winter prior to return and the previous summer for the four areas considered (electronic supplementary material, figure S2). Because mean SSTs in the different spatial aggregates are highly correlated (Pearson correlation greater than 0.7), we first identified the ocean areas for which the seasonal SST was most strongly correlated to the response variable (summer SST in the southeastern Bering Sea and winter SST in the area north and south of the Aleutian Islands). Fishery selection differentials in a given year and population were calculated as the difference between the mean size of fish in the escapement and the mean size of individuals in the total run, i.e. mean trait values after versus before selection [29]. This metric accounts for variation in harvest rate and selectivity and is typically used to estimate the strength of harvest selection (electronic supplementary material, figure S6). We used lags of about one generation to test for the strength of harvest selection on fish size, specifically 4- and 5-year lags as well as the average of the selection differentials in those 2 years. We allowed for nonlinear effects of all predictors by testing for quadratic terms and tested appropriate lags based on when predictors were hypothesized to affect sockeye salmon growth. We tested ocean temperatures and abundances of other salmon species in the 2 years prior to the sockeye salmon return to evaluate conditions during the years that both returning ocean age-2 and ocean age-3 fish experienced at sea. Because previous studies found that the relationship between temperature and salmon productivity changed with the 1988–1989 climate regime shift in the Northeast Pacific Ocean [30], we considered a factor variable representing the two regimes before and after 1988–1989 in interaction with SSTs. Pairwise correlations between predictors were computed and a Pearson correlation of 0.5 was used as a threshold for including any two predictors in the model (electronic supplementary material, table S1**)**. Because the time series of pink and chum salmon abundances were strongly correlated (Pearson correlation > 0.7), we included only pink salmon abundance, which consistently explained a higher proportion of the variance in the response compared to chum salmon abundance or their combined abundances. However, due to the strong correlation the effects of pink versus chum salmon cannot be fully distinguished statistically. Models were fit using multiple linear regression in R [31] assuming normally distributed errors. AIC-based model selection was performed using the dredge function of the package ‘MuMIn’ (v. 1.43.15, [32]). Details on the model selection procedure can be found in the electronic supplementary material.

## 3. Results

**Figure 2.**
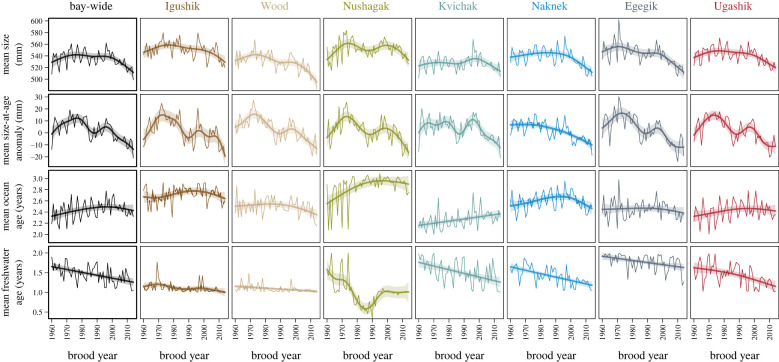
Demographic trends of Bristol Bay sockeye salmon. Changes in mean body size, mean size-at-age anomaly, mean ocean age and mean freshwater age (rows) bay-wide and for each river system (columns). Thin lines represent annual means and smoothers are nonlinear year effects on the annual means from generalized additive models with 95% confidence bands.

The mean body size of mature sockeye salmon returning to Bristol Bay increased during the 1960s brood years and remained relatively stable until the early 2000s brood years (figure 2). However, mean size subsequently declined such that fish are now returning at smaller mean sizes compared to any period during the 1960s to 2000s (figure 2). Mean body length decreased by about 19 mm or 3% bay-wide when comparing the most recent brood years (2010–2014; i.e. fish that returned to spawn in 2014–2020) to the early period (1960–1964). This change in mean body length translates into a decline in mean body mass of 10%, from an average of about 2.6 kg in the early period to about 2.3 kg in the most recent period. Most of this change occurred in the last decade of the dataset. A decreasing trend in the proportion of fish that spent 2 years in fresh water was apparent bay-wide (figure 2), especially the almost complete disappearance of 2.3 age fish in recent returns (electronic supplementary material, figure S5). Bay-wide age and size patterns are influenced by fluctuations in the relative abundance of fish returning to the different river systems, because each system exhibits characteristic age structures (electronic supplementary material, figure S5).

**Figure 3.**
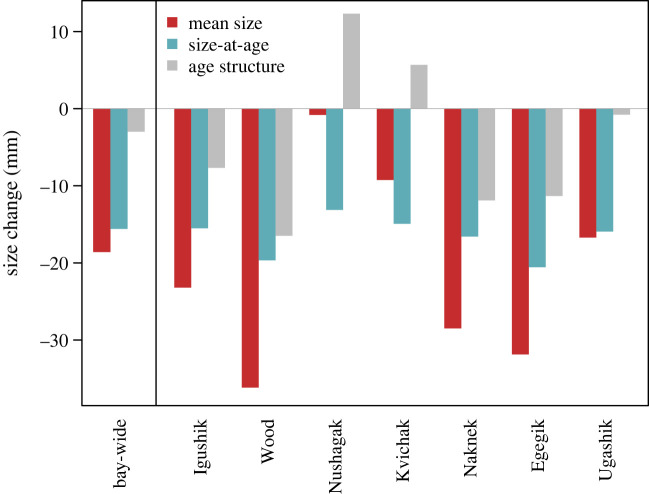
Contribution of trends in size-at-age to changes in mean body size of sockeye salmon. Estimated changes in mean body size (red) and mean size-at-age (blue) between the first (1960–1964) and last (2010–2014) 5 brood years of the time series for each river system and for all of Bristol Bay (bay-wide). The difference between changes in mean size and size-at-age is accounted for by shifting age structure (grey).

Declines in mean size varied considerably among river systems when comparing the most recent brood years (2010–2014) to the early period (1960–1964) (figure 3). The estimated size declines for the Igushik, Wood, Nushagak, Kvichak, Naknek, Egegik and Ugashik rivers were 23, 36, 1, 9, 28, 32 and 17 mm, respectively. Changes in size-at-age were more consistent among river systems (figure 2), with river-specific decreases in mean size-at-age from 13 to 21 mm and a bay-wide decline of 16 mm (figure 3). Changes in age structure over time were less consistent among rivers (figure 2) and contributions of shifting age structure to changes in mean size were highly variable (figure 3). The trends for more fish spending 3 years in the ocean, and thus returning at a larger size were partly compensatory to trends in decreasing size-at-age in two river systems (Kvichak and Nushagak rivers) for most of the early part of the time series. It should be noted, however, that the sockeye salmon population in the Nushagak River is distinct because it historically had larger proportions of river-type fish that have 0 freshwater age. Age composition in this system can change rapidly over time due to variation in the productivity of river versus lake-type sockeye salmon (e.g. Nushagak River freshwater age; figure 2). Bay-wide, trends in age structure accounted for only 3 mm of the decline in mean body size when changes up until the most recent brood years were evaluated (figure 3).

**Figure 4.**
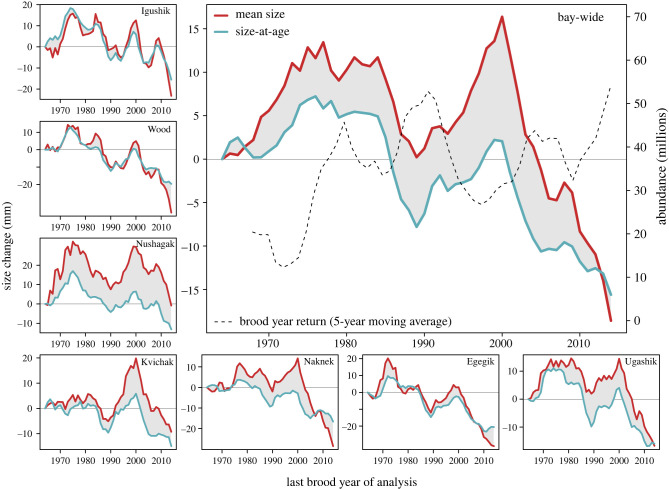
Retrospective analysis of the changing contribution of trends in size-at-age to changes in mean size of sockeye salmon. Shown are changes in mean size (red) and size-at-age (blue) for each brood year in the time series comparing the first and last 5 years of the respective period up to that brood year (e.g. when the last brood year of the analysis was 2000, the mean size for 1996–2000 was compared to that for 1960–1964).

Trends in age structure were at times compensatory to size-at-age declines at the bay-wide scale (mean age increased while size-at-age decreased thereby stabilizing the average size of fish in returns), especially during the mid-1980s to mid-1990s (figure 4). Bay-wide, mean size change was positive until the early 2000s brood years, and only declined thereafter, while size-at-age had already declined by the late 1980s. However, the temporal patterns of these variables differ considerably among river systems (figure 4). In some rivers, changes in mean size closely track changes in size-at-age, whereas in other rivers trends in age structure resulted in increased mean size over much of the time period. Importantly, several of the river-specific trends indicate that shifts in age structure are no longer compensatory and have thus become additive to trends in size-at-age, thereby accelerating the decline in mean body size. This accelerating trend became particularly pronounced at the bay-wide scale after brood year 2000, as evidenced by the rapidly declining mean size and size-at-age in return years beginning in about 2004.

**Figure 5.**
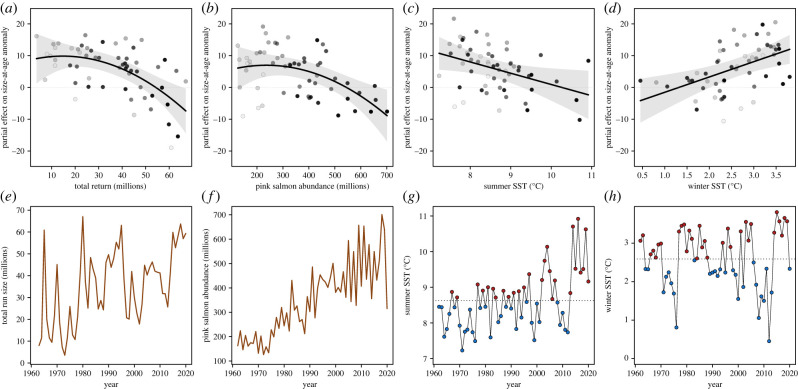
Estimated covariate effects on bay-wide size-at-age anomaly of sockeye salmon. Shown are partial effects (*a*–*d*) and time-series plots (*e*–*h*) of the model-selected predictors of mean size-at-age anomaly: return abundance of Bristol Bay sockeye salmon (*a,e*), abundance of pink salmon in the North Pacific (*b,f*), average summer SST in the Bering Sea (*c,g*) and average winter SST around the Aleutian Islands (*d,h*). Colours of circles in the top panels indicate the year (the lightest and darkest grey represent the first and last year of the time series, respectively), and polygons represent 95% confidence intervals. Colours of circles in the bottom panels (*g,h*) indicate SSTs above (red) and below (blue) the long-term average (dashed line).

**Figure 6.**
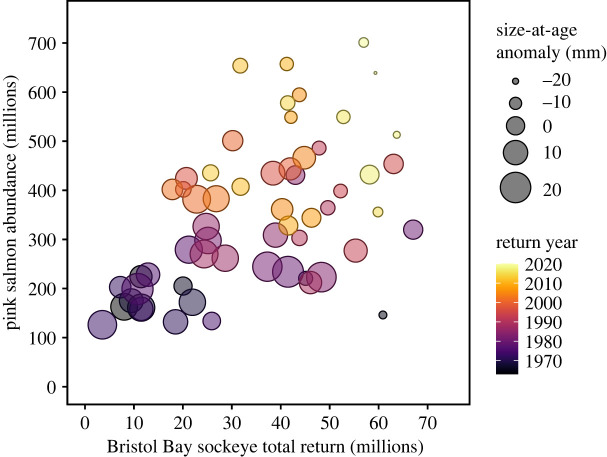
Size-at-age anomaly as a function of the return abundance of sockeye salmon and pink salmon abundance in the ocean in the previous year. Circle size indicates the size-at-age anomaly and colours indicate the year of return.

Annual variation in size-at age was largely explained by changes in salmon abundances and SST (figure 5). Variation in size-at-age was not associated with the strength of harvest selection on the parental generation. The best covariate model included the total return abundance of Bristol Bay sockeye salmon as well as the previous year's abundance of pink salmon in the North Pacific Ocean, mean winter SST around the Aleutian Islands and mean summer SST in the Bering Sea. These four covariates were included in all models with a *Δ*AIC < 2 (electronic supplementary material, table S2). Some of the alternative models with similar support did not include both quadratic terms for salmon abundances, and some included an interaction between SSTs and regime. Negative associations of size-at-age with the number of sockeye salmon returning to Bristol Bay and the abundance of pink salmon at sea suggest that both intraspecific and interspecific competition contribute to reduced growth rates. Furthermore, size-at-age was positively associated with winter SST, but negatively associated with summer SST, suggesting that warm summer temperatures result in slower growth in these populations. Parameter estimates and the equation of the selected model are presented in the electronic supplementary material, table S3. The four predictors included in the model explained about 64% of the variance in mean size-at-age anomaly and captured both interannual variability and long-term trends in size-at-age well (electronic supplementary material, figure S7). The return abundance of sockeye salmon, pink salmon abundance, and summer and winter SSTs explained 23.4, 24.9, 8.9 and 6.8% of the response variance, respectively. The size-at-age of Bristol Bay sockeye salmon was thus strongly associated with total sockeye salmon return abundance and the estimated abundance of pink salmon in the North Pacific Ocean (figure 6).

## 4. Discussion

Our analysis quantifies the contributions of shifting size-at-age and age structure to trends in overall mean size and reveals important mechanisms of declines in body size in the world's largest sockeye salmon fishery. We find that most of the decline in mean size of mature sockeye salmon has occurred since the early 2000s brood years. The size-at-age of sockeye salmon started to decline in the 1980s but mean sizes of fish in the returns were mostly compensated by fish spending longer in the ocean before spawning. Since the early 2000s, however, shifting age structure towards younger marine ages and declining size-at-age have acted in concert to cause significant declines in mean body size of mature sockeye salmon returning to Bristol Bay. Changes in size-at-age are strongly associated with increasing abundances of Bristol Bay sockeye salmon and other salmon in the ocean, suggesting competition as an important mechanism causing these size declines.

We show that changes in mean body size of Bristol Bay sockeye salmon have become apparent since the early 2000s brood years (for fish maturing 4–6 years later) because mean ocean age had increased previously and compensated for declines in size-at-age prior to the early 2000s. A recent study on size declines of Pacific salmon in Alaska concluded that declines in mean body size primarily resulted from changes in age structure [13]. In sockeye salmon, trends in size-at-age were estimated to explain 5.9% of changes in mean size since the mid-1970s, suggesting that salmon are getting smaller primarily because adults are returning to spawn at a younger age [13]. Their approach evaluated how year-to-year variation in mean size in return years, not long-term trends, was affected by shifting age structure and size-at-age. Their analysis was performed by return year, due to data limitations across species on a state-wide scale that precluded a more appropriate brood year analysis. Conducting analysis on return year data is problematic because diversity in age at maturity combined with recruitment variability leads to interannual variation in mean size at return even in the absence of demographic trends [28]. Strong contributions of changes in age proportions to interannual variation in mean size in return years are thus not necessarily indicative of strong contributions to long-term trends in mean size.

It has been suggested that long-term trends in the age-size structures of Pacific salmon in Alaska are linked to changes in climate and ecological interactions at sea [12,13,33]. Previous studies further indicated that fisheries may have caused body size declines in some populations and during times when size-selective harvest and exploitation rates were particularly high, but that fisheries are likely not a major driver of the broad-scale size declines observed in Pacific salmon [12,13,34,35]. In particular, a study on probabilistic maturation reaction norms of Bristol Bay sockeye salmon found that size-selective fishing may have contributed to reduced size-at-age of mature fish [36] while other work found little evidence that harvest intensity was linked to changes in sockeye salmon body size [13]. To test for potential effects of harvest selection on size-at-age, we used annual selection differentials in our model of changes in mean size-at-age anomaly, considering appropriate lags around one generation. The analysis suggested that harvest selection was not an important driver of changes in size-at-age. The Bristol Bay sockeye salmon fishery has generally been selective for larger fish, though selection patterns have varied over time and among rivers ([37]; electronic supplementary material, figures S5 and S9). Another factor that can contribute to changes in size-at-age over time is size-dependent survival, which can alter variability in phenotypic traits such as fish body size [38]. We calculated coefficients of variation in size-at-age by age group, which increased until the early 2000s but decreased thereafter (electronic supplementary material, figure S10), suggesting that size-dependent mortality is less important for causing changes in size-at-age compared to shifting growth rates. While we cannot rule out contributions of fishery-induced evolution or other sources of size-dependent survival to shifts in size at maturation, our results point to ecological changes, in particular competition at sea, as the major driver of changes in the size-at-age of Bristol Bay sockeye salmon. Changes in size-at-age of returning sockeye salmon further largely reflect altered growth during ocean residence, because growth potential is higher in the ocean compared to freshwater (electronic supplementary material, figure S8). Our results further suggest that competition in the ocean has negative effects on growth while the effect of temperature is season-specific, with warmer winters resulting in better growth, but warmer summers resulting in reduced growth.

Earlier work on Bristol Bay sockeye salmon showed that warming has led to shorter freshwater rearing of juveniles, and the earlier migration from freshwater to the ocean in combination with higher salmon abundances at sea has resulted in longer ocean residence [12]. Cline *et al*. [12] found that sockeye salmon that spent one rather than 2 years in freshwater were more likely to return after 3 rather than 2 years at sea, suggesting that a shorter freshwater residence increases the time sockeye feed in the ocean. Here we find that strong sockeye salmon returns and higher abundances of pink salmon in the ocean are also associated with reduced size-at-age, suggesting slower growth due to increased competition at sea. Negative associations between sockeye salmon body size and pink salmon abundance in the ocean [26,27], and the run size of sockeye salmon [39,40] have been reported previously. To what extent increasing numbers of chum salmon in the North Pacific Ocean have contributed to adverse effects of interspecific competition on sockeye salmon body size is difficult to disentangle due to the high correlation between the pink and chum salmon abundance time series. Return abundances of sockeye salmon to Bristol Bay have remained high in recent years. This apparent paradox of high returns despite intensified competition in the ocean may partially be explained by increased smolt production due to higher juvenile survival and/or relatively minor adverse effect on survival during early ocean residence. While competition has strong effects on growth during the second and third year at sea when salmon feed in offshore waters in the Gulf of Alaska [41], as suggested by our findings, marine survival is largely determined during the first year at sea [42], when Bristol Bay sockeye salmon mainly reside in the southeastern Bering Sea.

Pink and sockeye salmon show high diet overlap [43,44], indicating that direct competition for food might be causing the negative link between sockeye salmon size and pink salmon abundance. Previous research indicated that competition for food and density-dependent growth effects primarily occur when salmon feed in offshore waters [41]. Recent high seas salmon research in the central and eastern Gulf of Alaska suggests little spatial overlap between sockeye and pink salmon in winter/spring [45], yet knowledge about their spatial overlap in other areas and seasons is incomplete. These are important knowledge gaps that should be addressed in future research. Besides competition for shared prey, alternative explanations for the negative link between pink salmon abundance and sockeye salmon size include more complex effects via the food web that manifest as changes in growth opportunity for sockeye salmon or spatial–temporal avoidance of competitor species that restricts access to resources, all of which are based upon direct or indirect ecological interactions between the two species. However, some studies have indicated that food availability in the ocean may not be a limiting factor and that interspecific competition between salmon species may not be strong [46]. While conclusive experimental evidence for a phenomenon that plays out the scale of the North Pacific Ocean is not possible to obtain, (i) similar relationships have been reported between sockeye salmon, pink salmon and SST in other regions [47,48], (ii) we were unable to identify convincing alternative mechanisms to explain these negative relationships between body size and salmon abundances that do not involve direct or indirect ecological interactions within and between the two species, and (iii) the correlations between salmon abundances and sockeye salmon body size are strong [49]. The analyses presented here thus suggest significant negative effects of competition in the ocean on sockeye salmon body size. We found similar effects for the return abundance of Bristol Bay sockeye salmon and pink salmon abundance in the North Pacific, indicating that both intra- and interspecific competition contribute to variation in size-at-age. More research could improve understanding of the relative contribution of increasing abundances of chum salmon to changes in sockeye salmon body size.

A complex picture also emerges on how climate warming affects the growth, life-history and ultimately mean body size of sockeye salmon returning to Bristol Bay. Higher temperatures during freshwater residence support faster growth during the first year of life and hence earlier migration to the ocean [12]. This is the likely mechanism responsible for the proportional decline of the 2.2 and 2.3 age class fish (electronic supplementary material, figure S4). Once in the ocean, warmer winter temperatures increase growth and thus size-at-age, while warmer summer temperatures in the Bering Sea and Gulf of Alaska reduce sockeye salmon growth. Unusually warm summers have been observed in recent years in the North Pacific Ocean and the Eastern Bering Sea, especially during the 2014–2016 marine heatwave [50]. High SSTs presumably reduces size-at-age of Bristol Bay sockeye salmon because growth potential is limited by high metabolic demands or behavioural responses that result in lower food availability, such as occupying deeper waters, and other changes in the food base. Our finding that high summer SSTs (above approximately 10°C) can significantly reduce size-at-age is in line with experimental results on optimum growth temperatures for this species [51]. Experimentally determined optimum growth temperatures for sockeye salmon are around 15°C when excess food is available, but decline when food becomes limiting, to as little as 5°C for feeding rates of 1.5% of dry body weight per day [51]. While the experiments were conducted using juveniles, warm conditions that reduce growth may be reached at even lower temperatures in adult sockeye salmon, because optimal growth temperatures commonly decline with body size in fishes [52]. It is well known that the effects of increasing temperatures can differ between low- and high-competition environments, because warming-induced growth changes depend on food availability [53]. Whereas historical winter SSTs in the southern Bering Sea and northern Gulf of Alaska have been sub-optimal for Bristol Bay sockeye salmon, such that warmer winters tend to increase growth rates, summer SSTs appear to reach above-optimal temperatures in this population. Consequently, warming may lead to increased growth during colder seasons but reach high enough temperatures to reduce growth during extremely warm summers.

Declines in salmon body size have the potential to cause substantial losses to ecosystems and people as they reduce fish reproductive output and the economic value to fisheries [13]. On a per-fish basis, declines in body size translate into lower revenue to fishers and, in some markets, smaller fish demand a lower price per pound than large fish. Moreover, declines in the mean size of spawners have implications for fishery management because management goals for Pacific salmon are typically expressed in terms of the numerical abundance of fish on the spawning grounds, i.e. escapement goals. Spawner-recruit models that are used to inform escapement goals implicitly assume that each spawner contributes equally to recruitment, even though large fish, especially in females, have higher reproductive output [14,54]. Yet, return numbers of sockeye salmon to Bristol Bay are currently at a record high. How changes in demographic structure affect the long-term performance of current management remains unknown but should be considered when establishing spawner escapement goals that are adaptive to ongoing global change.

Record high returns of sockeye salmon to Bristol Bay during the last decade are associated with much of the observed size declines, emphasizing that there is a trade-off between fish abundance and body size. Bristol Bay provides one example of how fishery management and regulatory harvesting plans can accommodate changes in body size, because stock-specific escapement goal ranges are more precautionary than traditional escapement goals based solely on estimates of maximum sustained yield [55]. State regulations also provide guidance to managers to achieve higher range escapements in years with larger runs. Thus, during times of large runs, with smaller fish as shown here, a larger escapement is mandated by regulation. This management structure may better account for changes in fish size and reproductive potential. Adaptive in-season management in response to changing body size and the collection of relevant data may become more important when demographic changes are occurring at a rapid rate.

## Data Availability

Data and code are publicly available via GitHub at: https://github.com/janohlberger/SockeyeSize. The data are provided in the electronic supplementary material [56].
